# A Tailored Approach to Leishmaniases Vaccination: Comparative Evaluation of the Efficacy and Cross-Protection Capacity of DNA vs. Peptide-Based Vaccines in a Murine Model

**DOI:** 10.3390/ijms241512334

**Published:** 2023-08-02

**Authors:** Alicia Mas, Clara Hurtado-Morillas, Abel Martínez-Rodrigo, José A. Orden, Ricardo de la Fuente, Gustavo Domínguez-Bernal, Javier Carrión

**Affiliations:** 1 INMIVET Group, Animal Health Department, Veterinary School, Universidad Complutense Madrid, 28040 Madrid, Spain; 2 INMIVET Group, Animal Science Department, Veterinary School, Universidad Complutense Madrid, 28040 Madrid, Spain

**Keywords:** *Leishmania* vaccine, multi-epitope peptide vaccine, DNA vaccine, *Leishmania* immunology

## Abstract

Zoonotic leishmaniases are a worldwide public health problem for which the development of effective vaccines remains a challenge. A vaccine against leishmaniases must be safe and affordable and should induce cross-protection against the different disease-causing species. In this context, the DNA vaccine pHisAK70 has been demonstrated to induce, in a murine model, a resistant phenotype against *L. major*, *L. infantum,* and *L. amazonensis*. Moreover, a chimeric multiepitope peptide, HisDTC, has been obtained by in silico analysis from the histone proteins encoded in the DNA vaccine and has showed its ability to activate a potent CD4^+^ and CD8^+^ T-cell protective immune response in mice against *L. infantum* infection. In the present study, we evaluated the plasmid DNA vaccine pHisAK70 in comparison with the peptide HisDTC (with and without saponin) against *L. major* and *L. infantum* infection. Our preliminary results showed that both formulations were able to induce a potent cellular response leading to a decrease in parasite load against *L. infantum*. In addition, the DNA candidate was able to induce better lesion control in mice against *L. major*. These preliminary results indicate that both strategies are potentially effective candidates for leishmaniases control. Furthermore, it is important to carry out such comparative studies to elucidate which vaccine candidates are the most appropriate for further development.

## 1. Introduction

Cutaneous, muco-cutaneous, and visceral leishmaniosis are a group of diseases that occur due to an infection with different species of the protozoan parasite *Leishmania*. With a worldwide distribution, this vector-borne zoonosis results in different clinical manifestations, ranging from small skin ulcers to lethal systemic infection in viscerotropic species [1]. *Leishmania spp.* is transmitted by the bite of infected female sandflies from the genus *Phlebotomus* or *Lutzomyia*, depending on the area [2]. The disease is prevalent in more than 90 countries, and more than 1 billion people are living in endemic areas for leishmaniases [3]. Globally, leishmaniases are the third most important vector-borne diseases after malaria and lymphatic filariasis, being, in addition, a parasite that has infected more than 12 million people worldwide [4]. Over the past 20 years, efforts to control the disease have been made, with important advances in diagnosis and treatment; nevertheless, mortality and morbidity from leishmaniases still show a worrying increasing trend worldwide [5].

Cutaneous leishmaniosis (CL) is the most common form of leishmaniasis [6]. *Leishmania major* is one of the most important etiological agents of CL in the Old World. In addition, in immunocompromised hosts, it is associated with the progression to mucocutaneous leishmaniosis (MCL) [7]. On the other hand, *L. infantum* is responsible for most of the cases of visceral leishmaniosis (VL) in the Old World as well as in the New World [1]. VL is the most severe form of the disease, and, if no treatment is administered, may lead to death [8]. It is worth noting that *L. infantum* is capable of producing not only VL, but also CL. Both species are zoonotic, as *L. major* may use rodents as animal reservoirs, while *L. infantum* uses different mammals, such as dogs, cats, and hares, as reservoirs [9]. The infection of canids by *L. infantum* can lead to the development of canine leishmaniasis (CanL), which is a severe disease that may affect cutaneous, renal, ocular, skeletal muscle, and hemolymphatic target organs [10]. Furthermore, dogs are considered the main reservoir hosts of *L. infantum* for humans [11]. For these reasons, both *L. major* and *L. infantum* are of special public health relevance and require effective control measures to be developed.

Various therapeutic approaches are available for the control of leishmaniosis; nevertheless, the need for new and improved drugs is increasing due to the limitations associated with currently available treatments, including their potential toxicity, high cost, and reduced efficacy resulting from the emergence of drug resistance [12,13].

There are, at least as far as is recognized, 20 *Leishmania* species pathogenic to humans, most of which can also be transmitted to and cause established infections in other mammal species [2]. In this scenario, there is currently no vaccine available for humans [14]. On the contrary, in veterinary medicine, five vaccines against canine leishmaniosis have been licensed since 2004; nevertheless, doubts remain regarding their effectiveness, and only three remain on the market [15]. Recently, the pPAL-LACK vaccine has been approved by the European Medicines Agency (EMA) for commercialization by showing protection levels of approximately 60% in terms of parasite burden reduction in a murine model [16]. Overall, current available vaccines may decrease the possibility of developing clinical disease in canids but are not successful in entirely preventing the establishment of infection. Therefore, additional studies are needed for the evaluation of current vaccines and the development of future effective strategies against canine leishmaniosis [15,17].

In this context, the development of vaccine candidates that can provide cross-protection against different *Leishmania* species is of crucial importance. These vaccines should aim to elicit a robust immune cellular response capable of controlling parasite multiplication in target organs. This cellular response should be characterized by the elevated production of proinflammatory cytokines such as interferon-γ (IFN-γ) or interleukin-2 (IL-12) while minimizing the production of anti-inflammatory cytokines, such as IL-10 or IL-4 [18,19]. Having animal models that enable us to reproduce the disease is crucial for studying the immune response triggered by a potential vaccine candidate in a standardized manner. This allows us to compare its effectiveness with other strategies and analyze the immune mechanisms involved.

In the present study, we conducted a comparative analysis of different vaccine strategies [20,21] for the prevention of cutaneous and visceral leishmaniosis. The first strategy involved genetic vaccination using the plasmid pVAX1::HisAK70-asd (pHisAK70). The second strategy utilized a multi-epitope peptide called HisDTC alone or with saponin as an adjuvant. These strategies were evaluated using a well-established murine model of CL and VL caused by *L. major* and *L. infantum*, respectively.

The aim of this study was not only to compare, using the same conditions, different vaccination strategies, but also to present a murine model for the study of vaccination strategies against CL and VL caused by *L. major* and *L. infantum* that would allow us to characterize, in a standardized and reproducible way, the response of the animals in parasitological and immunological terms to different vaccine candidates.

## 2. Results

### 2.1. Immunizations using Both HisDTC w/o Saponin and pHisAK70 Induce Protection against VL and CL in Mice

The DNA plasmid pHisAK70 and the multiepitope peptide HisDTC with or without (*w*/*o*) saponin were used to immunize BALB/c mice three (pHisAK70) or two times (HisDTC) at 15 day intervals. After vaccination, animals were challenged with *L. major* or *L. infantum* in order to evaluate specific parasite control induced by immunization.

**Figure 1:**
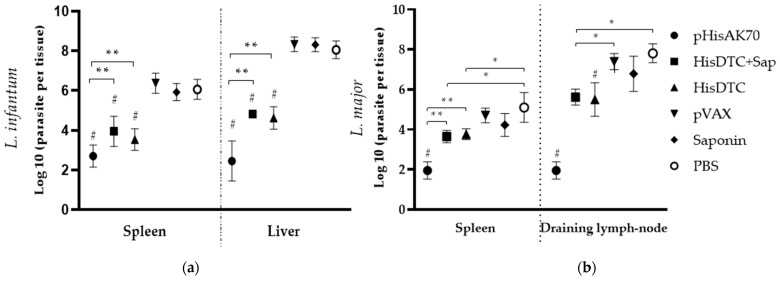
Parasite load in animals after in vivo infection. Six weeks after infection with *L. infantum* (**a**) and 7 weeks after infection with *L. major*, (**b**), animals were sacrificed, and the spleen and liver or draining lymph node were subjected to limiting dilution assay. Data are pre-sented as the mean ± SD. Hashes indicate significant differences (#, *p* < 0.001) between immunized (pHisAK70, HisDTC+Sap, and HisDTC) and control groups (pVAX, saponin, and PBS). Asterisks indicate differences (*, *p* < 0.05; **, *p* < 0.001).

To assess the effectiveness of controlling parasite multiplication, we quantified the parasite load in the target organs of the infected animals. As expected, all control groups (pVAX, saponin, and PBS) were unable to control the multiplication of both *L. infantum* (Figure 1a) and *L. major* (Figure 1b) in the target organs. In contrast, it could be observed that the group of animals immunized with the plasmid pHisAK70 achieved the best control of both *L. infantum* and *L. major* multiplication, presenting a statistically (*p* < 0.001) lower parasite load with at least 1 log difference in relation to the animals immunized with the peptide. In addition, animals immunized with the HisDTC peptide *w*/*o* saponin presented lower parasite load than the control groups.

**Figure 2:**
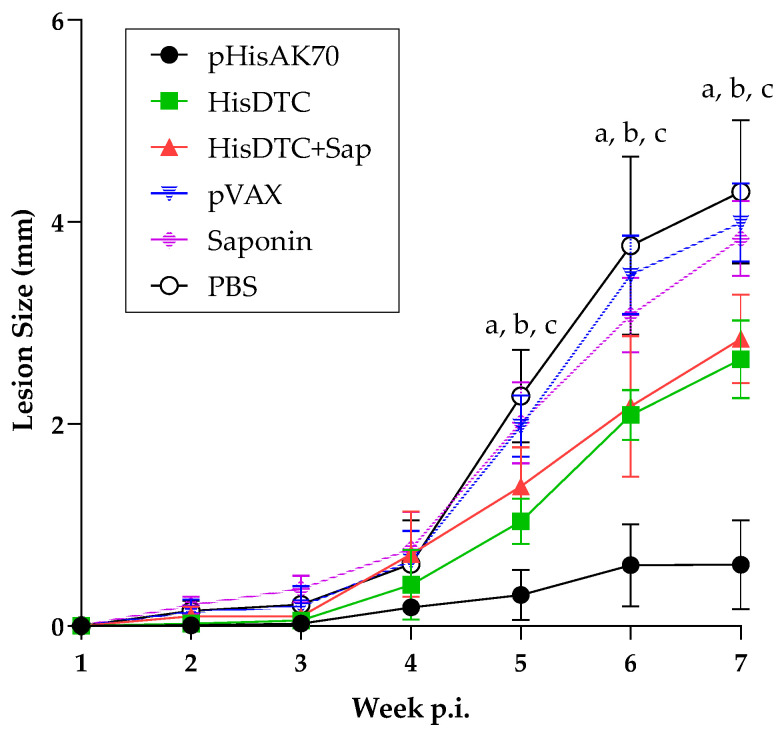
Lesion size of *L. major*-infected animals. Lesion size was measured by weekly evaluation during the infection. Data are presented as the mean ± S.D. Letters indicate differences (*p* < 0.05) between a—pHisAK70 group and the rest of the groups; b—HisDTC+Sap group and the rest of the groups except for the HisDTC group; and c—HisDTC group and the rest of the groups except for the HisDTC+Sap group.

Because *L. major* is a CL-producing species in murine models, we could study the development of the lesion size at the inoculation point in the footpad of the animals. In this way, we found that the immunized animals had a significant reduction in the size of the lesion compared to the animals corresponding to the control groups (Figure 2). In addition, footpad swelling was statistically smaller (*p* < 0.05) in the group immunized with pHisAK70 than the peptide-vaccinated animals, indicating that this strategy induced the best control in terms of developing the typical lesion produced by *L. major*.

Interestingly, it could be observed that the effect of saponin as an adjuvant to the multi-epitope peptide HisDTC, when evaluating the capability of the animals to control parasite multiplication, was non-existent, since there were no statistically significant differences between the HisDTC group and the HisDTC+Sap group in either the parasite loads in the target organs or the lesion size. This effect occurred in both *L. major*-infected and *L. infantum*-infected animals.

### 2.2. Immunization with the Different Strategies Promotes a Predominance of Cellular Immune Responses in Mice after Challenge

**Figure 3:**
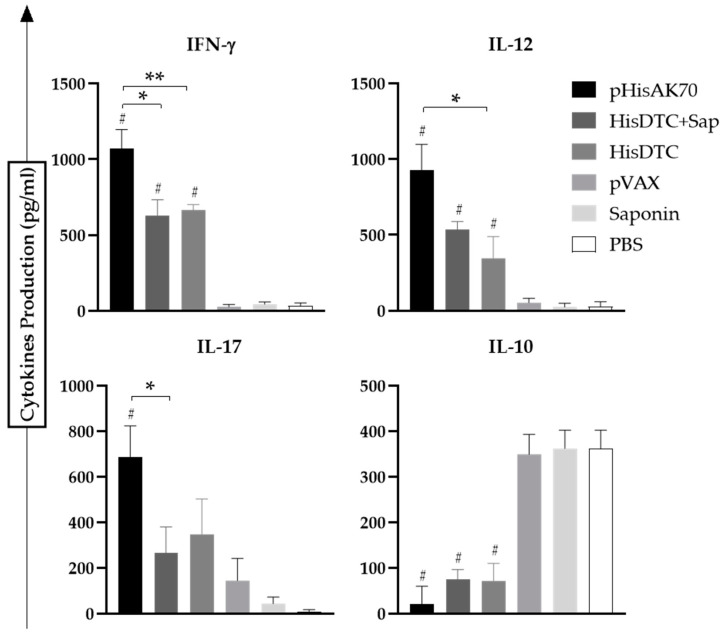
Cytokine production from the supernatant of the co-culture system of *L. infantum*-infected animals. Naïve BMDCs stimulated with specific SLAs were co-cultured with spleen cells from immunized and infected animals. ELISA was performed to quantify cytokine production. Data are presented as the mean ± SD. Hash marks indicate significant differences (#, *p* < 0.05) between immunized and control groups (pVAX, saponin, and PBS). Asterisks indicate significant differences (*, *p* < 0.05; **, *p* < 0.001).

To determine whether the immune phenotype of the vaccinated and infected animals was associated with a specific predominance of characteristic cytokines of the cellular immune response, supernatants from co-cultured cells of naïve bone-marrow-derived dendritic cells (BMDCs) stimulated using soluble *Leishmania* antigens (SLAs) with splenocytes of immunized and infected animals were evaluated. The pro-inflammatory (IFN-γ, IL-12, and IL-17) as well as anti-inflammatory (IL-10 or IL-4) cytokine production was quantified using ELISA kits. All vaccinated and *L. infantum*-infected animals presented statistically (*p* < 0.05) higher levels of specific IFN-γ and IL-12 when compared with control groups, thus indicating the ability of the pHisAK70 and HisDTC *w*/*o* saponin formulations to induce a predominance of proinflammatory cytokines characteristic of cellular immune response (Figure 3). Among the three vaccine formulations, the plasmid DNA was able to induce the highest production of both cytokines, which could be related to the greater control of parasite multiplication achieved in target organs. When considering the anti-inflammatory cytokines, it is worth noting that, in the VL model, all vaccine formulations were able to decrease with statistical significance (*p* < 0.05) the IL-10 production when compared to control groups. This Th1 cytokine bias towards IFN-γ/IL-12 pro-inflammatory cytokines was correlated with the IL-17 cytokine production, as previously described [22,23].

**Figure 4:**
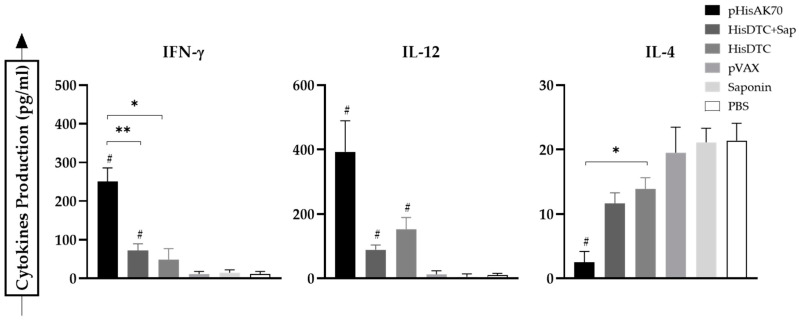
Cytokine production from the supernatant of the co-culture system of *L. major*-infected animals. Naïve BMDCs stimulated with specific SLAs were co-cultured with spleen cells from immunized and infected animals. ELISA was performed to quantify cytokine production. Data are presented as the mean ± SD. Hash marks indicate significant differences (#, *p* < 0.05) between immunized and control groups (pVAX, saponin, and PBS). Asterisks indicate significant differences (*, *p* < 0.05; **, *p* < 0.001).

In the case of infection with *L. major* (CL model), when analyzing the IL-4 production (Figure 4) as an indicator of Th2 or humoral response predominance, we could observe that only animals immunized with the pHisAK70 DNA plasmid produced less IL-4 than the control groups with statistical significance (*p* < 0.05). This IL-4 production was highly correlated with the lesion size control observed in these vaccinated animals. Similarly to what occurred in the case of *L. infantum*-infected animals, vaccinated animals infected with *L. major* were able to produce higher levels (*p* < 0.05) of IFN-γ and IL-12 in comparison with the control groups. Surprisingly, we could not find detectable levels of IL-17 in any animal infected with *L. major*.

**Figure 5:**
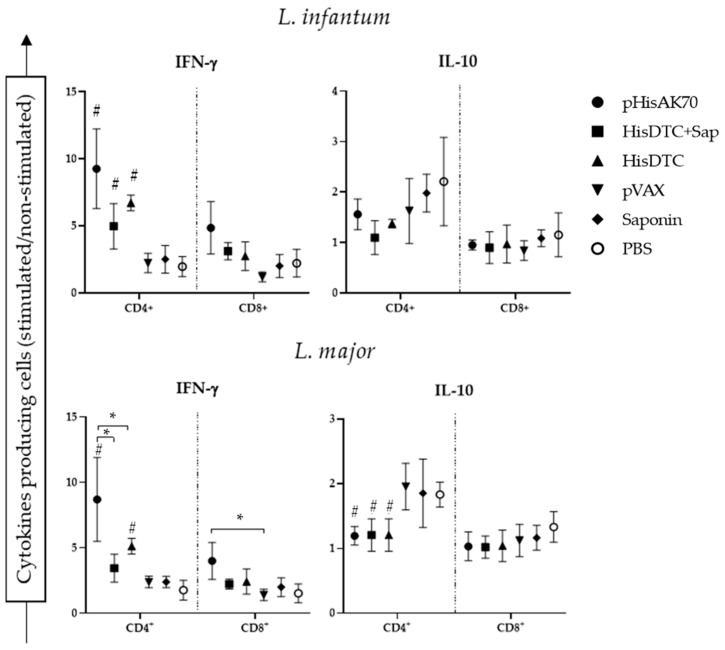
CD4^+^ and CD8^+^ T-cell involvement in the production of IFN-γ and IL-10. After sacrifice, some of the spleen cell suspensions were stimulated with specific *L. infantum* or *L. major* SLAs. The intracytoplasmic production of IFN-γ and IL-10 by CD4^+^ and CD8^+^ cells was measured by flow cytometry. Data are presented as the ratio between stimulated and non-stimulated cells. Data are presented as the mean ± SD. Hash marks indicate significant differences (#, *p* < 0.05) between immunized and control (pVAX, saponin, and PBS) groups. Asterisks indicate significant differences (*, *p* < 0.05).

The effective control of parasite multiplication in mice, thereby managing the progression of both *L. major* and *L. infantum* infections, relies on the activation of adaptive immunity [24]. In the murine model of VL and CL, the immune response is well-understood. A predominantly cellular response, mediated by IFN-γ CD4^+^- and CD8^+^-producing T helper cells (Th1), confers protection to the animals by clearing the parasites [25]. Conversely, a humoral immune response characterized by the production of anti-inflammatory cytokines, such as IL-10 in VL or IL-4 in CL, by CD4^+^ T helper cells (Th2) leads to susceptibility to infection [19]. Flow cytometry analyses of the splenic T-cell population producing IFN-γ and IL-10 revealed that vaccinated animals subsequently infected with *L. infantum* exhibited a higher specific population of IFN-γ producing CD4^+^ T cells compared to the control groups. No differences were detected between the pHisAK70 and HisDTC *w*/*o* saponin groups (Figure 5).

Furthermore, a similar trend was observed in animals that were immunized and challenged with *L. major*. Higher levels of intracytoplasmic IFN-γ CD4^+^ T cells were detected in the immunized compared to the control groups. In terms of cytokine production, as measured by the ELISA kit, animals immunized with pHisAK70 showed the highest percentage of IFN-γ CD4^+^ T cells (*p* < 0.05). When analyzing the population of IL-10 CD4^+^ cells, all immunized animals showed a lower percentage of this population compared to the control groups. However, no significant differences were observed among the vaccinated animals themselves. CD8^+^ T-cell-producing IFN-γ or IL-10 cytokines did not show any significant statistical difference between groups. This suggested that CD4^+^ T cells played a crucial role in generating the appropriate balance of cytokine profiles required for parasite control. Altogether, our results showed that the pHisAK70 vaccine induced a favorable Th1 phenotype, enabling the animal to control lesion size when infected with *L. major*. On the other hand, the HisDTC peptide with or without saponin demonstrated a similar effect to pHisAK70 in terms of cytokine balance when immunized animals were challenged with *L. infantum*.

### 2.3. Immunization with pHisAK70 Leads to the Development of Specific Immunological Memory Cells in Vaccinated Animals

The efficacy of vaccination is closely related to the ability of different vaccine strategies to modulate the adaptive immune response and generate memory cells that confer protection against reinfection. In the murine model, the spleen plays a crucial role in controlling systemic infections [26]. To assess the effectiveness of vaccination in stimulating and maintaining populations of memory T cells, we characterized and quantified the immune cell subsets in the spleen using flow cytometry analyses.

**Figure 6:**
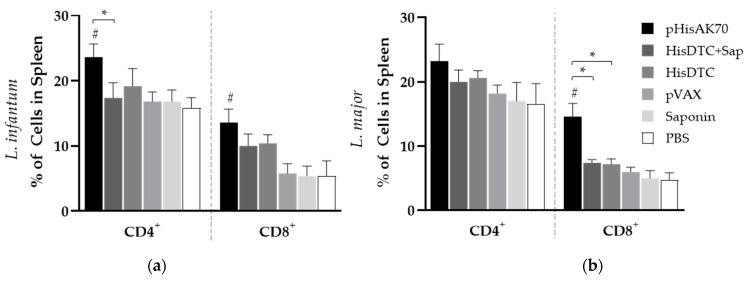
CD4^+^ and CD8^+^ T-cell population in spleens of *L. infantum-* (**a**) and *L. major-* (**b**) infected animals. After sacrifice, some spleen cell suspensions were stimulated with specific *L. infantum* or *L. major* SLAs. The percentage of live CD4^+^ and CD8^+^ cells were quantified by flow cytometry. Data are presented as the ratio between stimulated and non-stimulated cells. Data are presented as the mean ± SD. Hash marks indicate significant differences (#, *p* < 0.05) between immunized and control (pVAX, saponin, and PBS) groups. Asterisks indicate significant differences (*, *p* < 0.05).

Remarkably, only animals vaccinated with pHisAK70 exhibited an increased count of both CD4^+^ T and CD8^+^ T cells in the spleen when challenged with *L. infantum*, whereas, when infected with *L. major*, only an increment in the CD8^+^ T-cell population was observed (Figure 6).

**Figure 7:**
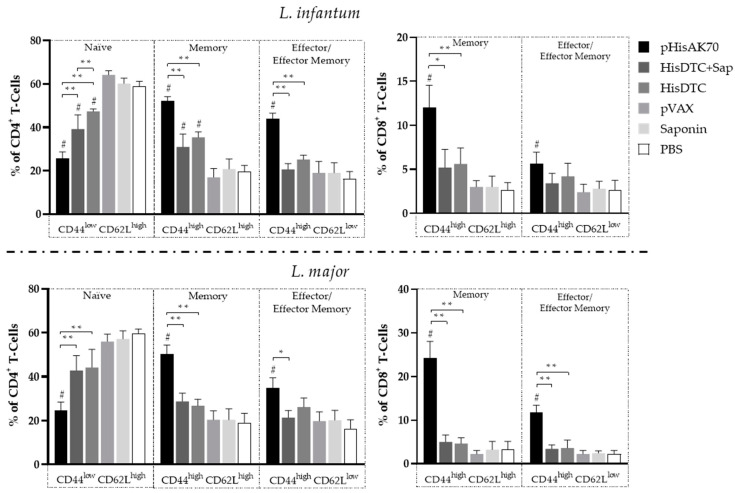
CD4^+^ and CD8^+^ memory and effector /effector memory T-cell subsets in spleens of *L. infantum-* and *L. major*-infected animals. After sacrifice, spleen cell suspensions were stimulated with specific *L. infantum* or *L. major* SLAs. The percentage of alive CD4^+^ CD44^+^ CD62L^+^ and CD8^+^ CD44^+^ CD62L^+^ cells was quantified by flow cytometry. Data are presented as the mean ± SD. Hash marks indicate significant differences (#, *p* < 0.05) between immunized and control (pVAX, saponin, and PBS) groups. Asterisks indicate significant differences (*, *p* < 0.05; **, *p* < 0.001).

Based on these findings, we decided to characterize the lymphocyte subpopulations among the different groups of animals (Figure 7). The data revealed that in animals vaccinated with pHisAK70 or HisDTC *w*/*o* saponin and subsequently infected with *L. infantum*, there was a statistically significant decrease (*p* < 0.05) in the population of naïve CD4^+^ T cells (CD44^Low^CD62^High^). Conversely, there was an increase in both memory (CD44^High^CD62L^High)^ and effector lymphocytes (CD44^High^CD62^Low^). Similarly, when examining the CD8^+^ T-cell subpopulations, an increase in the population of memory lymphocytes was observed, with statistical significance observed only in animals vaccinated with the DNA plasmid.

In the case of animals infected with *L. major* (Figure 7), a similar pattern was observed. When studying the evolution of the lesion on the paws of the animals, the group vaccinated with the pHisAK70 plasmid demonstrated the most pronounced bias in lymphocyte populations towards memory and specific effector T cells that were effective against the parasite. This effect was evident in both subpopulations of CD4^+^ and CD8^+^ T cells.

The results obtained indicated that all vaccination strategies were successful in generating a specific population of memory lymphocytes against *L. infantum*. Furthermore, the vaccine strategy utilizing the pHisAK70 plasmid exhibited the ability to generate a memory lymphocyte population that could also provide cross-protection against *L. major* species.

### 2.4. Immunization-Induce Protection in the Animals Is Reflected in the Modulation of Arginine Metabolism and Antibody Response

L-arginine is a crucial amino acid required for both the nitric oxide (NO)-mediated killing of parasites and the polyamine-mediated replication of parasites [27]. Due to these functions, a profile of resistance to infection was characterized by a predominant cellular immune response and the increased activity of the enzyme nitric oxide synthetase (iNOS) responsible for the production of NO. To determine the arginine metabolism in the infected animals, we employed a co-culture system consisting of SLA-stimulated BMDCs and splenocytes from immunized animals. In this system, we analyzed the production of arginine, which served as an indicator of failure in controlling parasite multiplication, as well as the production of nitrite, a derivative of NO, which served as an indicator of the leishmanicidal capacity of the animals.

The results obtained (Table 1) showed that the animals that were immunized and subsequently challenged with *L. infantum* produced a significantly higher (*p* < 0.05) amount of nitrites compared to the control groups. This indicated that these animals had a greater capacity to control the infection effectively. Additionally, it was observed that only the group vaccinated with pHisAK70 exhibited reduced levels of arginase, which was associated with the more effective control of the parasite induced by this vaccine strategy.

When we observed the results of the animals challenged with *L. major*, it was evident that all vaccinated groups exhibited a significant reduction (*p* < 0.05) in arginase production compared to the control groups. The decrease in arginase production was particularly pronounced in the animals vaccinated with pHisAK70. Importantly, in line with the findings obtained when analyzing lesion size and cytokine production, animals immunized with pHisAK70 were the only ones to demonstrate a higher level of nitrite production. This once again indicated that the pHisAK70 vaccination strategy was the most effective in controlling the multiplication of *L. major* in the animals.

**Figure 8:**
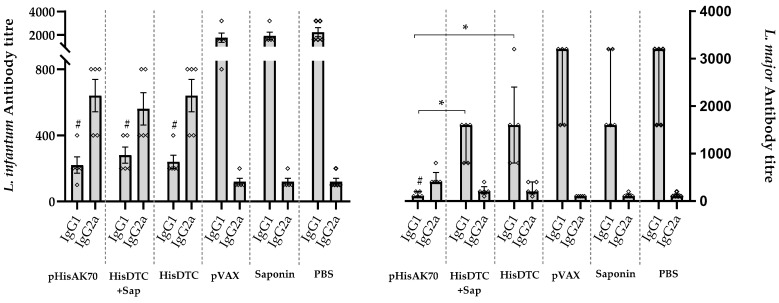
Humoral response after infection. Serum samples were collected from immunized and infected mice prior to sacrifice. The reactivity against specific SLAs was determined by evaluating the IgG1 and IgG2a isotype antibody levels of all animal groups. Data are presented as the median and the interquartile range of the reciprocal endpoint. Hashes indicate significant differences (#; *p* < 0.05) between immunized and control (pVAX, saponin, and PBS) groups. Asterisks indicate significant differences (*, *p* < 0.05).

Finally, following the identification of a reduction in parasite burden in target organs as a result of vaccination, we investigated the immune correlates of protection associated with immunoglobulin (Ig) production. To assess the humoral response in the animals, we quantified specific anti-SLA antibodies (Figure 8). In the case of animals infected with *L. infantum*, in the vaccinated groups, protection was correlated with a redirection of the IgG subclass toward the Th1-related IgG2a subclass of SLA-specific antibodies. Conversely, animals in the control groups (pVAX, saponin, and PBS) that were unable to control parasite multiplication exhibited predominantly Th2-related IgG1 subclass production. Regarding animals infected with *L. major*, the predominance of IgG2a antibody production over IgG1 was only detected in the pHisAK70 group, which was also correlated with a reduction in parasite burden.

## 3. Discussion

Over the years, a wide array of vaccine strategies have been evaluated against *Leishmania* parasites with different proven efficacies [14,28,29]. However, several factors have precluded the advancement of more than a few *Leishmania*-targeting vaccines into clinical trials [28,30]. Leishmaniases cover a wide range of clinical manifestations dependent upon the infecting parasite species. Each *Leishmania* species demonstrates a geographic range that is naturally determined by sand fly vectors, resulting in the presence of different forms of leishmaniases across endemic regions [2,31,32]. The relevance of these diseases emphasizes the importance of developing novel vaccine antigens and strategies capable of inducing long-lasting immune cross-protection against the various disease-causing *Leishmania* species.

In the present study, *L. infantum* and *L. major* were chosen as the target species due to their importance. *L. infantum* is a widely distributed species responsible for CanL and zoonotic VL, which is a life-threatening form of VL in humans. On the other hand, *L. major* causes CL, a debilitating human skin disease [1,33]. Moreover, these two species may coexist in the same region, such as Asia and the Middle East [34], and so when trying to design a vaccine, it is important that it is able to induce cross-protection for both species.

The efficacy of a vaccine relies on the immune response it generates, which can be characterized using an animal model that mimics the disease of interest. In this context, we decided to use the well-characterized BALB/c mouse model to evaluate various vaccine candidates against both *L. infantum* and *L. major* parasites [20]. Currently, numerous trials are underway to assess the effectiveness of different vaccine candidates [14]. However, it is crucial to conduct comparative trials under standardized conditions, allowing for the evaluation of different vaccine candidates and facilitating follow-up studies to identify the most promising strategies.

The present study aimed to evaluate and compare the efficacy of three different vaccine strategies in controlling *Leishmania* infection and eliciting a robust immune response against the parasite.

The animals in this study were vaccinated with two different vaccine candidates: the pHisAK70 plasmid and the HisDTC multiepitope chimeric peptide. These vaccine strategies were selected to represent the second- and third-generation vaccine approaches that are currently being studied in the field of leishmaniases. This encompasses protein/peptide-based vaccines, as well as DNA vaccines, which are promising avenues for developing effective vaccines against *Leishmania* infections.

The pHisAK70 plasmid, designated as pVAX1::HisAK70-asd, has previously been demonstrated to induce a protective immune response and efficacy against different *Leishmania* species in both murine and canine animal models [20,35,36]. The HisDTC peptide, when combined with saponin as an adjuvant, has been reported to generate a robust memory immune response based on the induction of a population of IFN-γ CD4^+^-producing T cells in a murine model of VL [21]. However, the currently licensed and marketed vaccine in Europe, LetiFend^®^, is a recombinant protein vaccine administered without adjuvants. In the present study, our goal was to evaluate the immunoprophylactic capacity of the HisDTC peptide in both the presence and absence of saponin, as well as to assess its cross-protective capacity against both *L. infantum* and *L. major* infections. Additionally, we compared the peptide-based vaccination strategy with the well-characterized pHisAK70 vaccine. This comparison allowed us to determine which vaccine induced the most effective immune response against the parasite and identify areas for improvement in each.

Based on our findings, the animals immunized with both plasmid DNA and peptide-based vaccines exhibited similar levels of reduction in parasite burden when challenged with *L. infantum*, regardless of the specific vaccine strategy employed. Surprisingly, the addition of saponin to the peptide did not significantly improve its effectiveness in reducing parasite burden. These results are consistent with those reported for the recently commercialized LetiFend^®^, which is a recombinant vaccine containing as the active substance a chimerical protein consisting of five antigenic fragments from four different *L. infantum* proteins utilized without adjuvants in a canine model of VL [37]. Furthermore, it is important to highlight that advances in the use of system biology to probe the molecular networks driving immune response to vaccines (system vaccinology) have revealed mechanistic insights for the development and design of vaccine and adjuvant strategies [38].

Recent advances in understanding leishmaniasis progression have revealed that cellular interactions extend beyond the conventional Th1/Th2 paradigm and play a crucial role in determining the course of infection. One important cell population may be Th17 cells, which primarily influence neutrophil recruitment and play a dual role at the site of infection [39]. The observed reduction in the parasite burden found in the vaccinated animals and infected with *L. infantum* may be attributed to the generation of IFN-γ, IL-12, and IL-17, as detected in the co-culture supernatant of naïve BMDCs in contact with splenocytes from immunized and infected animals. Consistent with the existing literature, our data indicated that, although pHisAK70-immunized animals were the only ones able to produce statistically higher levels of IL-17, all immunized animals were capable of producing a higher amount of this cytokine compared with the control groups, which was correlated with the subsequent production of IFN-γ and a decrease in the production of immunosuppressive cytokines such as IL-10 [22,23]. Importantly, the animals that demonstrated more effective control of parasite multiplication in target organs were those that produced higher amounts of Th1 characteristic cytokines. Conversely, the control groups failed to effectively control the infection and exhibited the elevated production of Th2-characteristic cytokines such as IL-10.

We also analyzed the specific CD4^+^ and CD8^+^ T-cell population responsible for the production of IFN-γ and IL-10. Surprisingly, animals that were vaccinated and subsequently infected presented a significant increase in the CD4^+^ T-cell population producing IFN-γ, with even higher levels observed in animals immunized with pHisAK70. However, no notable differences were observed in the CD8^+^ T-cell population. These findings highlighted the critical role of CD4^+^ T cells in disease control, which aligns with previous studies demonstrating their importance in effectively controlling *Leishmania* multiplication [40,41]. Moreover, it has been shown for other vaccine candidates [42] that the depletion of CD8^+^ T cells producing IFN-γ in vaccinated animals is correlated with a significant loss of protection in the target organs, as we observed in the control groups.

Vaccination with pHisAK70 and the HisDTC multiepitope chimeric peptide *w*/*o* saponin confirmed the antigenicity of both formulations. When animals were immunized and subsequently challenged with *L. infantum*, we observed the differentiation of certain CD4^+^ T-cell-specific subpopulations, namely central memory (CD44^High^CD62L^High^) and effector/effector memory (CD44^High^CD62L^Low^) T cells. These subpopulations are known to play a key role in establishing a long-lasting effective immune response against *Leishmania* parasites [43,44,45,46]. Moreover, immunized animals presenting specific memory and effector CD4^+^ T cells against *L. infantum* also demonstrated the higher production of Th1 characteristic cytokines. This indicated the induction of an antigen-specific Th1 immune response related to parasite control. Furthermore, it is well-known that the leishmanicidal mechanism, which involves the production of NO, is stimulated by IFN-γ-producing CD4^+^ T cells [47]. In this regard, our immunized animals that were able to reduce the parasite load in the target organs also presented a higher level of iNOS activation in proportion to IFN- γ production. Additionally, immunization with pHisAK70 induced a memory (CD44^High^CD62L^High^) and effector/effector memory (CD44^High^CD62L^Low^) CD8^+^ T-cell subset. Consistent with our results, previous studies have described the importance of CD8^+^ T cells in eliminating *Leishmania*, as animals that exhibit better control of parasite multiplication tend to have a larger population of CD8^+^ T cells [48].

Altogether, our results indicated that all vaccine strategies employed in this study were effective in significantly reducing *L. infantum* parasite multiplication in target organs by establishing a specific Th1 immune response. However, animals immunized with pHisAK70 appeared to exhibit better control of the disease in terms of parasite load, cytokine production, and the induction of memory and effector T cells. Moreover, similarly to what has been described, when the animals were vaccinated and subsequently challenged with *L. major*, all vaccinated animals were capable of controlling parasite multiplication at the site of infection. However, it is noteworthy that in the case of *L. major* infection, the differences between animals immunized with the pHisAK70 plasmid and those immunized with the peptide *w*/*o* saponin were more pronounced. On the contrary, the response to the multi-epitope HisDTC chimera was milder, resulting in larger lesion sizes compared to the plasmid-based vaccine. These findings were consistent with the observed parasite loads, indicating a correlation between the immune response elicited by the vaccines and the control of parasite multiplication.

The HisDTC peptide had not been previously evaluated against a CL model. This peptide is based on highly conserved *Leishmania* proteins (histones H2a, H2b, H3, and H4). However, although the selected epitopes generated a protective response in animals immunized and infected with *L. infantum*, the response appeared to be less effective against *L. major*. It is important to highlight that these findings regarding parasite load were correlated with the cytokine production observed in these animals, since animals with a smaller lesion size (immunized with pHisAK70) showed a higher production of cytokines characteristic of a Th1-type response (IFN-γ and IL-12) and the lower production of IL-4. In CL-causing species, increased IL-4 production has been demonstrated to be associated with susceptibility to infection in mice [19].

As shown in the animals infected with *L. infantum*, all vaccination strategies elicited in animals infected with *L. major* the differentiation of CD4^+^ T-cell subpopulations, including specific central memory (CD44^High^CD62L^High^) and effector/effector memory T cells (CD44^High^CD62L^Low^). However, only the pHisAK70 strategy was capable of inducing a memory (CD44^High^CD62L^High^) and effector/effector memory (CD44^High^CD62L^Low^) CD8^+^ T-cell subset. The implication of the CD8^+^ T-cell-mediated killing of infected cells is not fully understood, as it may contribute to parasite elimination, or, if exacerbated, lead to cytotoxicity-induced pathology [48]. Our findings showed that the presence of the memory and effector CD8^+^ T-cell subset in the murine model of CL caused by *L. major* was associated with a smaller lesion size at the inoculation site and the control of parasite multiplication in target organs. These findings were also correlated with the CD8^+^ T cells producing IFN-γ, as only the animals capable of better controlling disease progression, i.e., the pHisAK70-vaccinated group, presented a higher percentage of this specific T-cell population. Remarkably, our investigations did not reveal detectable levels of IL-17 in any of the animals infected with *L. major*. This unexpected outcome prompts further exploration into the underlying mechanisms and potential implications of the absence of IL-17 in this context. As expected, animals vaccinated with pHisAK70 presented the higher activation of the iNOS enzyme, as evidenced by nitrite production, which was correlated with the higher production of IFN-γ. Although all vaccinated animals presented a smaller lesion size compared to the control groups, it was well-established that pHisAK70 was the strategy that most effectively induced parasite control, as shown by the reduced parasite load in target organs, cytokine production, and the induction of memory and effector T-cell responses.

Taken together, our results highlighted the variable efficacy of different vaccine strategies in controlling the multiplication of both *L. infantum* and *L. major* in target organs. The use of standardized animal models is crucial for accurately comparing and evaluating different vaccine candidates. This approach enabled us to identify the strengths and weaknesses of each of the strategies, providing valuable insights for the improvement and optimization of future vaccine development efforts.

## 4. Materials and Methods

### 4.1. Mice, Parasites, and Preparation of Soluble Antigens

Eight-week-old female BALB/c mice (Janvier-Labs, Laval, France) were maintained under specific pathogen-free conditions. The study was approved by the Animal Welfare Committee of the Community of Madrid, Spain, (reference: PROEX 211/18) following Spanish and EU legislation (Law 32/2007, R.D. 53/2013, and Council Directive 2010/63/EU).

*L. infantum* (M/CAN/ES/96/BCN150 zymodeme MON-1) and *L. major* (clone V1: MHOM/IL/80/Friedlin) were cultured as previously described [49] at 26 °C in Schneider′s medium (Sigma-Aldrich, Saint Louis, MO, USA) supplemented with 20% of inactivated fetal bovine serum (FBS, Sigma-Aldrich, Saint Louis, MO, USA); 200 U/mL penicillin; and 200 mg/mL streptomycin (Lonza, Basel, Switzerland).

Specific soluble *Leishmania* antigen (SLA) was prepared from stationary-phase promastigotes, as described in [50]. Specific SLA obtained from *L. infantum* or *L. major* was used depending on the species employed for the animal challenge.

### 4.2. Vaccine Preparation

The DNA vaccine pVAX1::HisAK70-asd (pHisAK70) and the empty vector pVAX1-asd (pVAX) were previously constructed as described in [20]. Briefly, the DNA plasmids were purified using an EndoFree Plasmid Giga Kit (Qiagen, Hilden, Germany) according to the manufacturer’s instructions. The endotoxin-free DNA plasmids were resuspended in sterile saline solution and stored at −20 °C until use.

The multiepitope peptide vaccine HisDTC was previously designed in [21]. The chimeric peptide was synthesized by GenScript Biotech (Leiden, The Netherlands) with a purity ≥ 95%. The synthetic multiepitope peptide was dissolved in Dulbecco’s phosphate-buffered saline (PBS) (Sigma-Aldrich, Saint Louis, MO, USA) according to the hydrophobicity. The HisDTC peptide was stored at −20 °C until use. Saponin (*Quillaja saponaria*) was used as an adjuvant when needed.

### 4.3. Immunization Protocol and Infection

Immunizations were carried out in 6 groups (*n* = 10/group), as follows: vaccinated groups—pHisAK70, HisDCT with saponin, and HisDTC without saponin; control groups—pVAX, saponin and PBS control group. The animals were subcutaneously immunized with 175 µg of the pHisAK70 plasmid or the empty vector pVAX, or with 50 µg of HisDTC peptide *w*/*o* 25 µg of saponin for the HisDTC+Sap or HisDTC groups, respectively. The saponin group was inoculated with 25 µg of saponin alone. The PBS group was inoculated with PBS by the same procedure. The DNA plasmid pVAX1:HisAK70-asd (pHisAK70) and the multiepitope peptide HisDTC previously diluted in PBS with and without saponin were used to immunize BALB/c mice 3 or 2 times at 15 day intervals, respectively.

To evaluate the efficacy of the different vaccine strategies against VL, four weeks after the last immunization, half of the animals of each group were intravenously injected with 5 × 10^5^ *L. infantum* stationary-phase promastigotes. To analyze the effectiveness of vaccination in the murine model of CL, four weeks after immunization, animals were inoculated S.C. in the left footpad with 10^4^ metacyclic *L. major* promastigotes in a volume of 30 µL. Metacyclic promastigotes (infective-stage) were isolated from stationary-phase cultures by negative selection using peanut agglutinin (Vector Laboratories, Newark, CA, USA), as previously describe [51].

### 4.4. Parasite Burden, Lesion Size, and Antibody Production after Infection with L. infantum and L. major

To perform parasitological and immunological analyses, six weeks after *L. infantum* inoculation, animals were sacrificed. For animals inoculated with *L. major*, the course of infection was monitored weekly by measuring footpad swelling thickness with a metric caliper and expressed as the increase in the thickness of the infected footpad compared to the uninfected footpad. Seven weeks post-infection, mice were euthanized as the lesion size of the control groups was higher than 4 mm in diameter, which was a previously established clinical endpoint criterion.

Once animals were sacrificed, the sera, spleen, and liver or draining lymph node were collected. To evaluate the parasite load, infected tissues were subjected to a limiting dilution assay. Briefly, tissues were homogenized using a glass tissue grinder in 4 mL of complete Schneider’s medium. A limited dilution assay was performed as described elsewhere [52,53]. Two hundred microliters were plated into 96-well flat-bottom microtiter plates (Nunclon, Thermo Fisher Scientific, Waltham, MA, USA) and diluted in log-fold serial dilutions using the same medium. After 10 days being maintained at 26 °C, samples were analyzed by optical microscopy. The results are expressed as the log of the number of parasites in each organ calculated from the reciprocal of the highest dilution containing viable promastigotes.

For the antibody production assay, blood samples were collected from the animals before euthanasia. Standard endpoint ELISA was performed as previously described [52] to determine specific *L. infantum* or *L. major* anti-SLA antibodies (Abs). Briefly, 96-well flat-bottomed microtiter plates (Nunc Immunoplate, Maxisorb, Merck, Darmstadt, Germany) were coated overnight at 4 °C with 100 μL of SLA (8 μg/mL) diluted in PBS. Negative and positive control sera were obtained from parasite-free and *L. infantum-* or *L. major*-infected mice, respectively. Serum samples were serially twofold diluted starting from 1/100. All samples were analyzed individually. Peroxidase-labeled goat anti-mouse IgG isotypes (IgG1 and IgG2a, dilution 1/8000, Sigma-Aldrich, St. Louis, MO, USA) were used as secondary Abs. The enzyme-labeled complexes were detected by reactions with the TMB substrate. The reaction was stopped with 50 μL of 2 M sulfuric acid after 15 min of reaction according to the manufacturer’s instructions, and the optical density was read using a spectrophotometer at 450 nm (BenchMark Plus, Bio-Rad Laboratories, Hercules, CA, USA). The reciprocal endpoint titer was defined as the inverse value of the highest serum dilution factor, providing an absorbance three times higher than the negative control.

### 4.5. Generation of Bone Marrow Dendritic Cells

Bone marrow stem cell progenitors were obtained from the femurs and tibiae of naïve BALB/c mice (*n* = 3) and cultured in complete medium (CM) consisting of RPMI (1640 with L-glutamine, Lonza, Basel, Switzerland) supplemented with 10% FBS; a mixture of antibiotics (100 U/mL penicillin, 100 mg/mL streptomycin, Lonza); and 10 mM HEPES (Lonza) in the presence of 20 ng/mL murine granulocyte-macrophage colony-stimulating factor (GM-CSF; PeproTech, London, UK), as previously described [54]. Fresh medium containing GM-CSF was added to the cultures every 3 days. On day 7, half of the volume was removed, and cells were centrifuged at 500× *g* for 10 min. The pellet was then resuspended on CM with freshly added growth factor and cultured at 37 °C with 5% CO_2_. On day 10, nonadherent cells were collected and considered BMDCs based on the expression of CD11c as described before [20].

### 4.6. Cytokine Production after Splenocyte Stimulations

With the aim of analyzing the cytokine levels induced in vaccinated and infected animals, naïve BMDCs were seeded (10^6^ cells/mL) into 24-well plates and pulsed overnight with 25 µg/mL of specific *L. infantum* or *L. major* SLAs. Subsequently, BMDCs were incubated in the presence of splenocytes isolated from the spleen of immunized and infected mice at a 1:5 ratio (BMDCs:splenocytes), as previously described [21]. Supernatants were collected after 96 h, and IFN-γ, IL-12 (p40), IL-17, and IL-10 or IL-4 levels were measured using commercial ELISA kits following the manufacturer’s instructions, as follows: for IFN-γ, the Eli-pair kit (Diaclone, Besanços, France); for IL-12 (p40) and IL-10, the BD OptEIA kit (Bioscience, San Diego, CA, USA); and for IL-4 and IL-17, the Duoset ELISA kit (Development System R&D, Abingdon, UK).

In parallel, the cell response was also evaluated by flow cytometry. For this purpose, spleen cells (5 × 10^6^ cells) from immunized and infected animals were collected and stimulated in vitro in polypropylene tubes (Falcon, Heidelberg, Germany) with specific *L. infantum* or *L. major* SLAs (25 μg/mL) for 48 h at 37 °C in 5% CO_2_, whereas the non-stimulated culture only received medium. The intracytoplasmic IFN-γ (Clone XMG1.2, Biolegend, San Diego, CA, USA) and IL-10 (Clone JES5-16E3, Biolegend)-producing T-cell profile was measured as described elsewhere [55] in both CD4^+^ cells (Clone GK1.5, Biolegend) and CD8^+^ cells (Clone 53-6.7, Biolegend). Dead cells were excluded using a LIVE/DEAD Zombie NIR Fixable Viability Kit (Biolegend). Briefly, the measurements were performed on a CytoFLEX^®^ instrument (Beckman Coulter, Life Science, Indianapolis, Indiana, Brea, CA, USA), and the CytExpert™ software package (Beckman Coulter, Life Science, Indianapolis, IN, USA) was used for analysis based on 100,000 events per sample. The percentage of specific cytokine-producing-CD4^+^ or CD8^+^ T cells relative to the total number of CD4^+^ or CD8^+^ T cells was determined by the analysis of FACS data using CytExpert™ software. The results are expressed as indexes that were obtained by comparing the ratios of CD4^+^ and CD8^+^ T cells in the SLA-stimulated cultures to the values obtained for the non-stimulated cells (ratio: stimulated culture/non-stimulated culture).

### 4.7. Enzyme Modulation of Vaccinated Animals after Infection

The L-arginine metabolism was studied by the arginase and nitric oxide-synthetase activity. As described before, naïve BMDCs were pulsed with specific SLAs and co-cultured with splenocytes of immunized and infected animals at 37 °C, 5% CO_2_, for 96 h. Supernatants were collected, and the concentration of nitrites, which are a byproduct of nitric oxide (NO) production, was measured using the Griess reaction, as described in [56]. Subsequently, the cells were incubated for 30 min in lysis buffer (0.1 M Tris–HCl, pH 7.5, 300 μM NaCl, 1 μM PMSF, 1% Triton X-100), and lysates were assayed for intracellular arginase activity, as previously described [57]. One unit of enzyme activity was expressed as the amount of enzyme that catalyzes the formation of 1 mmol of urea/min.

### 4.8. Analysis of the Memory and Cellular Immune Response in the Spleen by Flow Cytometry

The establishment of long-lasting immunity and the subsequent protection against *Leishmania* parasites is dependent on the production of specific memory and effector T cells. To analyze the lymphocyte population, spleens from vaccinated and infected animals were obtained. Briefly, splenocytes were processed as described below to obtain a single-cell suspension. Subsequently, cells were counted, and 10^6^ cells/mL were incubated in the presence of 25 μg/mL of specific *L. infantum* or *L. major* SLAs at 37 °C, 5% CO_2_ for 24 h. Cells were then triple-stained with anti-CD4 FITC (Clone GK1.5, Biolegend) or anti-CD8 FITC (Clone 53-6.7, Biolegend) and anti-CD44 (Clone IM7, Biolegend) and anti-CD62L (Clone MEL-14, Biolegend) for 30 min extracellular staining at 4 °C in the dark. Cells were then fixed with paraformaldehyde 4% *w*/*w*. Dead cells were excluded using a LIVE/ DEAD Zombie NIR Fixable Viability Kit (Biolegend). Cells were analyzed on a CytoFLEX^®^ instrument (Beckman Coulter, Life Science, Indianapolis, IN, USA), and the CytExpert™ software package version 2.5 (Beckman Coulter, Life Science, Indianapolis, IN, USA) was used for analysis based on at least 100,000 events per sample.

### 4.9. Statistical Analysis

Data are presented as the mean ± standard deviation (SD) and as the median and the interquartile range in the case of the antibody response. The statistical analyses were performed using GraphPad Prism software (version 8.3 for Windows, San Diego, CA, USA). To assess normal distribution, we performed the Shapiro–Wilk normality test; then, analyses were conducted using one-way ANOVA with the multiple-range Tukey’s test to determine which means from the independent groups were significantly different. Differences between immunized and control groups are shown with hashes, and differences between groups are shown with asterisks. The antibody response of animals was analyzed using the Kruskal–Wallis test, as these data did not follow a normal distribution. Differences were considered significant when the *p*-value ≤ 0.05.

## 5. Conclusions

In conclusion, the present study emphasized the importance of using standardized animal models to compare and evaluate different vaccine candidates for leishmaniases. Among the evaluated strategies, the DNA plasmid-based pHisAK70 vaccine showed promise as a candidate capable of inducing cross-protection against multiple *Leishmania* species. Its potential for protecting both *L. infantum* and *L. major* makes it a valuable tool in the fight against leishmaniases. Furthermore, the novel use of the HisDTC peptide in the absence of an adjuvant demonstrated the significant immunoprophylactic potential for controlling zoonotic VL caused by *L. infantum*. Moreover, the HisDTC peptide *w*/*o* saponin demonstrates that, despite it being capable of inducing a predominant Th1 immune phenotype in animals that were vaccinated and then infected with both *L.infantum* and *L. major*, the parasite multiplication was higher when vaccinated animals faced *L. infantum*. This opens up possibilities for the use of novel delivery systems that can enhance the immune response observed in this study. The findings shed light on the effectiveness of different vaccine strategies against *L. infantum* and *L. major* infection and provide insights for future research and the development of effective vaccines against leishmaniases. However, further studies are necessary to optimize vaccine strategies and assess their applicability in real-world settings for controlling this complex disease. Continued research and development are crucial to advance our understanding and combat leishmaniases effectively.

## 6. Patents

The HisDTC peptide is covered by a patent license (EP4163303A1). This patent is the property of the Universidad Complutense de Madrid, Spain.

## Figures and Tables

**Table 1:** Enzymatic activity of BMDCs in a co-culture system with spleen cells from vaccinated and infected animals.

	Infected by *L. infantum*		Infected by *L. major*	
Groups:	mU Arginase Activity	µM Nitrites	mU Arginase Activity	µM Nitrites
**pHisAK70**	6.25 ± 2.34 ^#, a^	31.68 ± 7.03 ^#^	3.85 ± 078 ^#, a, b^	21.75 ± 3.80 *
**HisDTC+Sap**	12.65 ± 1.59	15.74 ± 2.75 ^#^	20.90 ± 1.59 ^#^	6.64 ± 2.06
**HisDTC**	14.13 ± 3.67	17.58 ± 3.37 ^#^	18.78 ± 3.67 ^#^	8.78 ± 1.50
**VAX**	19.82 ± 3.00	5.73 ± 3.05	47.28 ± 3.00	2.96 ± 1.33
**Saponin**	22.20 ± 10.62	4.81 ± 2.62	52.50 ± 11.38	2.86 ± 1.95
**Control**	21.06 ± 6.20	4.34 ± 2.44	64.39 ± 6.20	1.94 ± 1.37

Data are presented as the mean ± SD. Hash marks indicate significant differences (#, *p* < 0.05) between immunized and control (pVAX, saponin, and PBS) groups. Asterisks indicate significant differences (*, *p* < 0.05) with all other groups. The letter a indicates significant differences (^a^, *p* < 0.05) with the HisDTC+Sap group. The letter b indicates significant differences (^b^, *p* < 0.05) with the HisDTC group.

## Data Availability

No new data, other than that already presented in the manuscript, were created or analyzed in this study.
